# Visualisation of limb movements by accelerometers in sedated patients

**DOI:** 10.1186/s13054-020-02975-7

**Published:** 2020-06-03

**Authors:** Erlend Flinstad Harbo, Silje S. Fuglerud, Nils Kristian Skjærvold

**Affiliations:** 1 Department of Circulation and Medical Imaging, Norwegian University of Science and Technology, Trondheim, Norway; 2 Department of Electronic Systems, Norwegian University of Science and Technology, Trondheim, Norway; 3 Department of Endocrinology, Trondheim University Hospital, Trondheim, Norway; 4 Department of Anaesthesia and Intensive Care Medicine, Trondheim University Hospital, Trondheim, Norway

Dear Editor,

The prognostication of neurological outcome in sedated ICU patients is challenging. Multiple clinical scoring schemes and examinations are used, where different motoric responses are important input variables. Accelerometery is a well-known technology widely applied in different fields of research and everyday electronic products. In medical research, accelerometers have been used in longitudinal epidemiological studies of physical activity and health as well as in ICU studies on the topic of activity, sleep and agitation monitoring. Similarly, accelerometric information could be a candidate to improve future neurological prognostication schemes. To the best of our knowledge, including a systematic review from 2015 [1], there are no published articles on automatic motion registration from ICU patients in connection to neurologic outcome prognostication.

We investigated a small population expected to experience motoric changes over a limited time period. After institutional approval and patient consent, we connected four wireless 3D accelerometric AX3 sensors (Axivity Ltd., Newcastle, UK) to the limbs of 10 post-cardiac surgery patients in the cardiothoracic ICU while still in general anaesthesia. We collected accelerometric data and observed their limb movements as the sedation was pre-described and the patients woke up. Movement artefacts induced by the health personnel were kept at an absolute minimum.

The raw data sampled at 100 Hz mandates processing before providing sensible information. The three-dimensional acceleration vector for each extremity was combined into one according to

$$ A=\sqrt{x^2+{y}^2+{z}^2}-1. $$

A peak finder function was applied with height 50*σ* + *μ*, where *σ* was the standard deviation and *μ* the mean of the first stabilising period when the patient was fully immobile, set to 2 min. The time-distance of the function was set to 50 samples. The acceleration peaks were summed within 1-min epochs to visualise the movement data as heat maps. This greatly reduces the amount of data that the clinician encounters and eases the interpretation. Finally, all data were truncated to 10 min starting at least 2 min before the first detectable movement. All analyses were performed in the Python programming language [2]. An example of the stages of data and graphical processing is shown in Fig. 1.

**Fig. 1:**
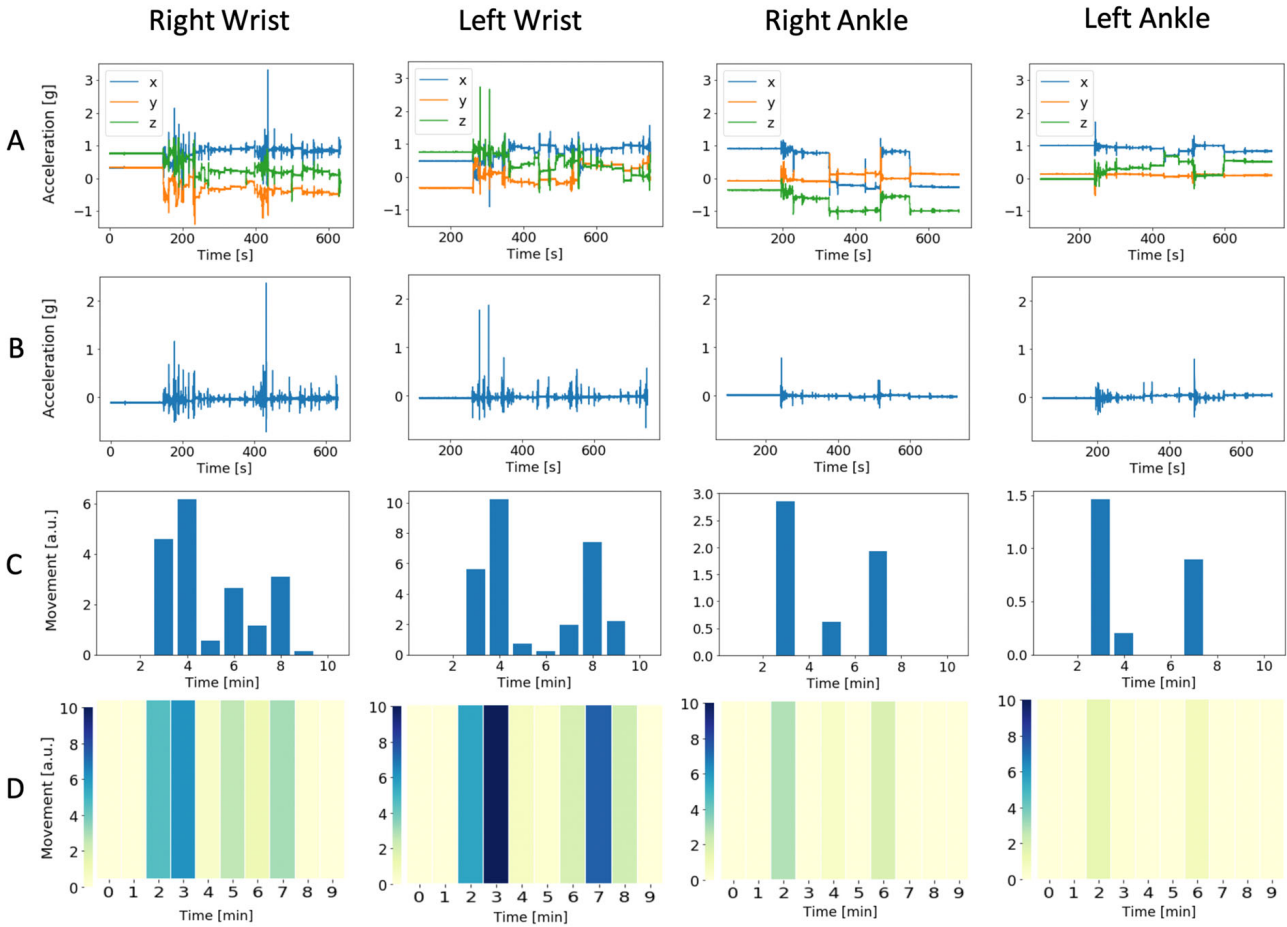
Example of the stages of data and graphical processing from one study subject, one column for each of the four limbs and the rows according to **a** raw accelerometric data in three dimensions, **b** 3D data condensed to one variable per limb, **c** total amount of limb movement shown as histograms in 1-min epochs, and **d** heat plot of the data from **c**

In all patients, this approach detected a change from no limb movements to various degrees of movement in one or several limbs, as shown in Fig. 2. Since the time from de-prescription until wake-up varies substantially between individuals, we highlighted the 10 min with most movements from each patient, including at least a 2-min start period of baseline no movement. Interestingly, all patients went into a motionless period after the wake-up period, visible in the figure. The quantity of movement correlated with our manual observations and was highly individual.

**Fig. 2:**
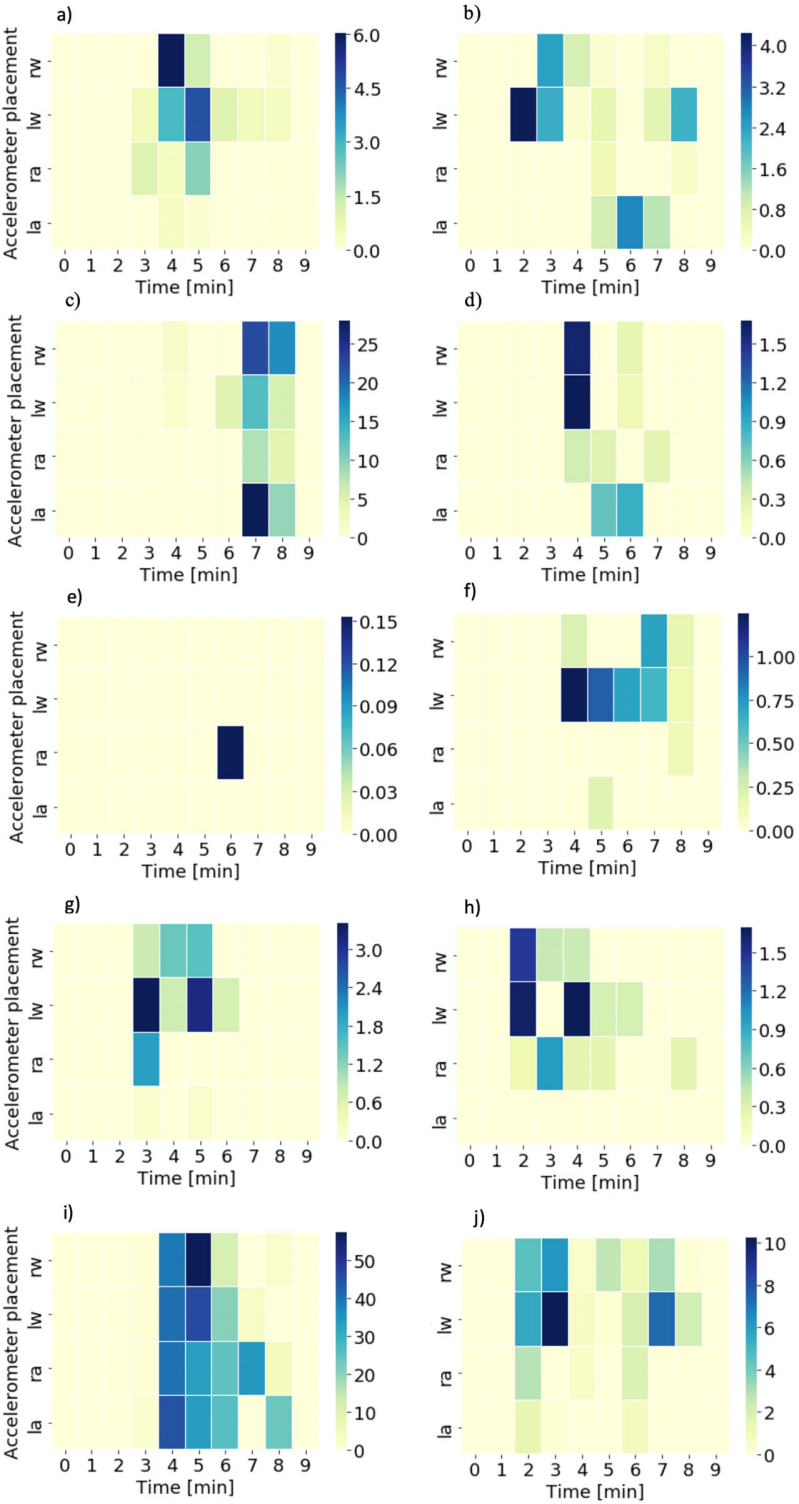
Heat map of limb movements in all 10 patients (**a** to **j**) with the amount of movement for each limb as a function of 1-min time epochs over 10 min. la left ankle, ra right ankle, lw left wrist, rw right wrist

We show the possibility of detecting the amount of movements in sedated patients and to present these data in a simplified manner to the clinician. As such, we are expanding the use and usability of accelerometric data from earlier studies which mainly focused on sedation and agitation levels [3, 4]. In ICU patients, there are several known types of abnormal movements depending on the underlying pathophysiology of brain injury [5, 6]. In this first study, we did not aim at doing any qualitative movement characterisation albeit we do believe this should be possible with the use of accelerometers in the future utilising the full time-resolution of the sensors. It will, however, require more complex data processing algorithms made from larger, labelled datasets including both accelerometric data and clinically validated brain injury diagnosis.

Limitations to this trial are the low number of participants and the choice to study post-surgery patients and not the designated user population of critically ill patients with a potential brain injury.

## Data Availability

The datasets used and/or analysed during the current study are available from the corresponding author on reasonable request.

## References

[1] Verceles AC (2015). Use of accelerometry to monitor physical activity in critically ill subjects: a systematic review. Respir Care.

[2] Welcome to Python.org. Python.org. Available from: https://www.python.org/about/. [cited 2020 Feb 24].

[3] Raj R, Ussavarungsi K, Nugent K (2014). Accelerometer-based devices can be used to monitor sedation/agitation in the intensive care unit. J Crit Care.

[4] Kamdar BB, Kadden DJ, Vangala S, Elashoff DA, Ong MK, Martin JL (2017). Feasibility of continuous actigraphy in patients in a medical intensive care unit. Am J Crit Care.

[5] Hannawi Y, Abers MS, Geocadin RG, Mirski MA. Abnormal movements in critical care patients with brain injury: a diagnostic approach. Crit Care. 2016;20(1):60. 10.1186/s13054-016-1236-2 PMC4791928 26975183

[6] Freund B, Kaplan PW (2017). Myoclonus after cardiac arrest: where do we go from here?. Epilepsy Curr.

